# Female Infertility Associated with Blood Lead and Cadmium Levels

**DOI:** 10.3390/ijerph17051794

**Published:** 2020-03-10

**Authors:** Sohyae Lee, Jin-young Min, Kyoung-bok Min

**Affiliations:** 1 Department of Preventive Medicine, College of Medicine, Seoul National University, Seoul 03080, Korea; leesohyae@snu.ac.kr; 2 Institute of Health and Environment, Seoul National University, Seoul 08826, Korea; yaemin00@snu.ac.kr

**Keywords:** reproductive health, female infertility, heavy metals, lead, cadmium, cross-sectional study

## Abstract

Lead and cadmium are known to be potential female reproductive toxins. However, studies on the relationship between these metals and infertility are limited. This study examines the association between self-reported infertility and blood lead and cadmium levels in US women by comparing metal levels in infertile and pregnant women. Data on blood lead, blood cadmium, and infertility from women aged 20–39 years who participated in the 2013–2014 and 2015–2016 National Health and Nutrition Examination Surveys were analyzed (*n* = 124, ‘pregnant’ *n* = 42, ‘infertile’ *n* = 82). Blood lead and cadmium levels were measured using inductively coupled plasma mass spectrometry, and infertility and pregnancy status were assessed using a self-reported questionnaire. Low blood lead and cadmium levels (geometric mean of blood lead = 0.50 µg/dL and blood cadmium = 0.26 µg/L) were positively associated with self-reported infertility after adjusting for confounding effects (odds ratio (OR) for lead per two-fold increase in blood metal levels = 2.60; 95% confidence interval (95% CI), 1.05–6.41 and OR for cadmium per two-fold increase = 1.84; 95% CI, 1.07–3.15). Although our findings require confirmation, they suggest that even low blood cadmium and lead levels may be deleterious to female fecundity.

## 1. Introduction

Infertility, which is commonly defined as no pregnancy after one year of unprotected intercourse, affects millions of couples worldwide [1,2]. In the US, approximately 7.4% [2] to 15.5% [3] of couples are affected by infertility. However, the utilization of infertility services remains low at 12.0% [4]. Unexplained etiology, which accounts for 8–28% of all infertility cases [5], is caused by various lifestyle (i.e., age, nutrition, exercise, obesity, psychological stress, smoking, and alcohol use) and occupational factors (i.e., shift work, stress, and physical agents) [6]. Furthermore, an important risk factor for infertility is exposure to environmental pollutants such as chemicals, air pollution, and heavy metals [7,8].

Lead and cadmium are ubiquitous heavy metals that are known to be potential female reproductive toxins [9]. Exposure to lead has been associated with menstrual cycle disturbances, low gestational weight, premature birth, and miscarriages [10]. Animal studies have provided relatively convincing evidence on the relationship between lead and infertility and have shown the negative effects of lead on fertility, folliculogenesis, luteal function, and pituitary membrane function [11,12,13,14]. However, only a few human studies support such findings [15,16,17,18]. Elevated blood lead levels have been found in infertile women [17] and have been linked to prolonged time-to-pregnancy [16]. Nevertheless, other studies have reported that there is no association between lead exposure and female fecundity [19,20,21]. The association between cadmium and infertility has been studied to a lesser extent. Experimental data have revealed that exposure to cadmium affects female fertility by altering ovulation, steroidogenesis, pituitary function, and fertilization [8,22,23,24]. Although only a few human studies have been conducted on this topic, females with high exposure to cadmium in the workplace [18] or those residing near a lead-zinc mine had higher risks of conception delay, idiopathic infertility, and difficulty conceiving [25]. Conversely, conflicting results have been observed regarding the association between blood cadmium levels and fecundity [17,19].

Despite the effect of cadmium and lead exposures on female reproduction in animals, studies on these relationships are limited and conflicting in humans. Although the basis behind such inconsistent outcomes is not readily apparent, factors such as small sample sizes and the inclusion of participants in hospital settings (i.e., from fertility clinics) may contribute to such inconsistencies [17,19]. Up until now, there have been no population-based studies examining these effects. Accordingly, in the present study, we aimed to assess whether blood lead and cadmium levels were associated with self-reported infertility in US women by comparing blood lead and cadmium levels between infertile and pregnant women.

## 2. Materials and Methods

### 2.1. Study Population

Data on lead, cadmium, and infertility were obtained from the 2013–2014 and 2015–2016 National Health and Nutrition Examination Surveys (NHANES), a nationally representative survey of the non-institutionalized civilian population in the US [26]. A total of 10,251 women were included from both cycles. Women who were pregnant at the time of interview were included in the ‘pregnant’ category (*n* = 118) regardless of a history of infertility (among the 118 pregnant women, 15 women reported to have a history of infertility), and non-pregnant women with a self-reported history of infertility were included in the ‘infertile’ category (*n* = 238). The analysis was restricted to sexually experienced women (‘pregnant’ *n* = 114, ‘infertile’ *n* = 234) aged 20–39 years (‘pregnant’ *n* = 108, ‘infertile’ *n* = 158) without a history of hysterectomy and/or bilateral oophorectomy (‘pregnant’ *n* = 108, ‘infertile’ *n* = 157). A one-half sample from participants aged 12 years and older were eligible for blood lead and cadmium sampling (‘pregnant’ *n* = 42, ‘infertile’ *n* = 82). Among these subjects, 124 women had data for all covariate variables.

### 2.2. Self-Reported Infertility

Infertility is defined as the absence of pregnancy with unprotected intercourse for one year [1]. The presence of infertility was assessed using a self-reported questionnaire. The prevalence of infertility among women aged 20 years and older was assessed using the following question: “Have you ever attempted to become pregnant over a period of at least a year without becoming pregnant?” Women who replied “yes” were identified as infertile.

### 2.3. Measurements of Blood Lead and Cadmium Levels

Whole blood specimens (with a minimum volume of 0.25 mL) were frozen (−30 °C) before being shipped to the National Center for Environmental Health and the Centers for Disease Control and Prevention, Atlanta, GA for testing. Blood lead and cadmium levels were measured using an inductively coupled plasma mass spectrometer with dynamic reaction cell technology (ELAN^®^ DRC II; PerkinElmer Norwalk, CT, USA) after a simple dilution sample preparation step. All reported results met the extensive quality control criteria outlined by the Division of Environmental Health Laboratory Sciences [27,28]. The lower limit of detection (LLOD) was 0.07 µg/dL for blood lead and 0.1 µg/L for blood cadmium in the 2013–2016 NHANES. For concentrations below the LLOD, a value equal to the LLOD divided by a square root of 2 was used [29,30].

### 2.4. Other Variables of Interest

Variables of interest were obtained from the interview data of the 2013–2016 NHANES and included age (20–24, 25–29, 30–34, or 35–39 years), ethnicity (non-Hispanic white, non-Hispanic black, Hispanic, or other), annual family income (less than $20,000 or $20,000 or more), education (less than high school, high school graduate, or more than high school), marital status (married, never married, widowed, divorced, separated, or living with partner), smoking status (never, ex-smoker, or current smoker), alcohol consumption (yes or no), physical activity (yes or no), and body mass index (BMI) (underweight (<18.5 kg/m^2^), normal weight (18.5–24.9 kg/m^2^), overweight (25.0–29.9 kg/m^2^), or obese (>30 kg/m^2^)).

### 2.5. Statistical Analysis

Statistical differences in demographics and health behaviors between the two groups (infertile vs. pregnant) were evaluated using the chi-squared test. Blood concentrations of lead and cadmium were analyzed as both continuous and categorical variables. Distributions of blood levels of lead and cadmium were right-skewed and log transformed. Blood levels of lead and cadmium were also categorized into tertiles. Blood lead levels ranging from 0.11 µg/dL to 0.38 µg/dL were categorized as tertile 1 (T1), 0.41–0.62 µg/dL as T2, and 0.63–5.37 µg/dL as T3. Blood cadmium levels between 0.07 µg/L and 0.19 µg/L were categorized as T1, 0.20–0.33 µg/L as T2, and 0.34–5.14 µg/L as T3. Geometric means were used. The Cochran–Armitage test for trend was used to assess the presence of an association between reproductive status (infertility and pregnancy) and blood metal tertiles (lead and cadmium).

To evaluate the association between blood metals and reproductive status, logistic regression analyses were performed using the SURVEY procedure. We calculated the odds ratio (OR) of the correspondence between reproductive status and blood metal levels and its 95% confidence interval (CI) and compared each tertile of the lead or cadmium distribution with the lowest tertile of exposure (reference group). The regression models were adjusted for age, ethnicity, annual family income, education, marital status, smoking history, alcohol consumption, physical activity, and BMI.

According to the NHANES analytic and reporting guidelines, all analyses applied weighted estimates of the population parameters to account for the complex sampling design. SAS 9.4 statistical analysis package (SAS Institute, Cary, NC, USA) was used, and the statistical significance was set at α = 0.05.

## 3. Results

### 3.1. Characteristics of Study Participants

Table 1 shows the sociodemographic characteristics of study participants. The overall prevalence of infertility in this sample was 66.13% (82 cases), whereas pregnant women comprised 33.87% (42 cases) of the sample. The infertile women were more likely to be older. There were no statistically significant differences in ethnicity, annual family income, education, marital status, smoking history, alcohol consumption, physical activity, and BMI between the infertile and pregnant women. The geometric means (95% CI) of the blood lead and cadmium levels for the sample of 124 women were 0.50 µg/dL (95% CI: 0.43, 0.57) and 0.26 µg/L (95% CI: 0.22, 0.31), respectively.

### 3.2. Number of Events (Infertility and Pregnancy) According to Blood Lead and Cadmium Tertiles

**Figure 1:**
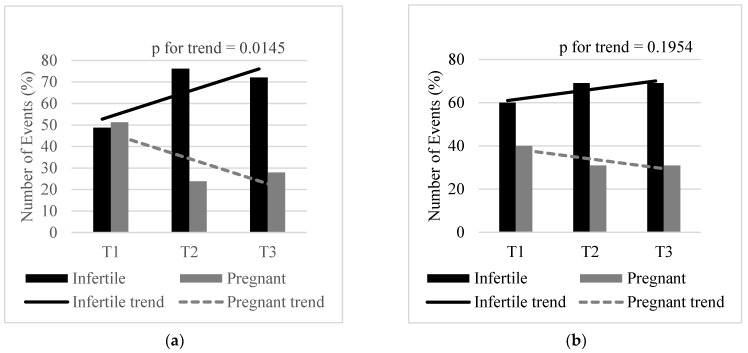
Number of events by blood lead (**a**) and blood cadmium (**b**) tertiles. Lead T1: Tertile 1 (0.11–0.38 µg/dL), T2: Tertile 2 (0.41–0.62 µg/dL), T3: Tertile 3 (0.63–5.37 µg/dL). Cadmium T1: Tertile 1 (0.07–0.19 µg/L), T2: Tertile 2 (0.20–0.33 µg/L), T3: Tertile 3 (0.34–5.14 µg/L).

Figure 1 shows the number of events (infertility and pregnancy) according to blood lead (Figure 1a) and blood cadmium (Figure 1b) tertiles. The proportion of infertile to pregnant women shows an increase from the first to second tertile (0.49:0.51 vs. 0.76:0.24) and a decrease from the second to third tertile (0.76:0.24 vs. 0.72:0.28) (Table 2). Overall, the number of infertile women showed a statistically significant increasing trend with increasing blood lead tertiles (*p* = 0.0145). Although there was an increase in the proportion of infertile to pregnant women from the first to second and third blood cadmium tertiles (0.60:0.40 vs. 0.69:0.31 vs. 0.69:0.31), no statistically significant trend was observed (*p* = 0.1954) (Table 2).

### 3.3. Blood Metal Levels and Reproductive Status

Table 2 shows the association between blood metal levels and the odds of being infertile. Unadjusted ORs revealed that a two-fold increase in blood lead, but not cadmium, concentrations was associated with infertility with an OR (95% CI) of 2.06 (1.32–3.23) and 1.18 (0.83–1.66), respectively. Unadjusted ORs for infertility, comparing blood lead tertiles 2 and 3 with the lowest tertile, were 3.99 (95% CI: 1.60, 9.99) and 3.08 (95% CI: 1.26, 7.54), respectively. Corresponding adjusted ORs for blood cadmium were 1.19 (95% CI: 0.35, 4.09) and 1.59 (95% CI: 0.60, 4.26), respectively. After adjusting for age, ethnicity, annual family income, education, marital status, smoking history, alcohol consumption, physical activity, and BMI, we found that a two-fold increase in blood lead and cadmium concentrations were significantly associated with infertility, with an adjusted OR (95% CI) of 2.60 (1.05–6.41) and 1.84 (1.07–3.15), respectively. Fully adjusted ORs for infertility, comparing blood lead tertiles 2 and 3 with the lowest tertile, were 5.40 (95% CI: 1.47, 19.78) and 5.62 (95% CI: 1.13, 27.90), respectively. Corresponding adjusted ORs for blood cadmium were 1.15 (95% CI: 0.22, 5.90) and 2.47 (95% CI: 0.6, 10.17), respectively. Fully adjusted ORs comparing tertiles 2 and 3 of blood lead and cadmium with the lowest tertile revealed a dose–response relationship.

### 3.4. Adjusted Means of Blood Lead and Blood Cadmium Levels

**Figure 2:**
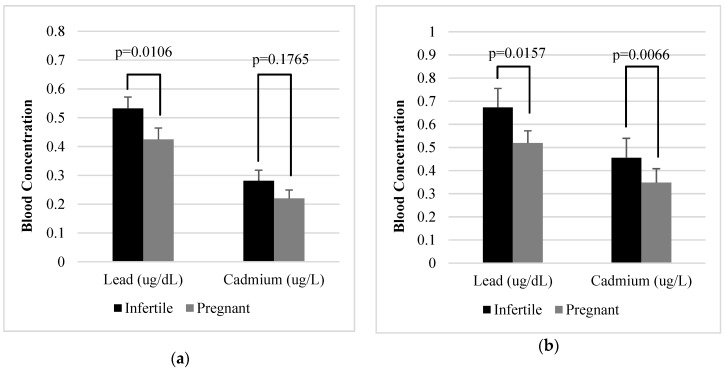
Age-adjusted means (**a**) and all-adjusted means (**b**) of blood lead and blood cadmium levels.

Women in the infertile group had higher mean blood lead levels (0.55 µg/dL, 95% CI: 0.47, 0.64) and cadmium levels (0.27 µg/L, 95% CI: 0.22, 0.34) than women in the pregnant group (mean lead: 0.40 µg/dL, 95% CI: 0.34, 0.48; mean cadmium: 0.23 µg/L, 95% CI: 0.17, 0.31); however, a statistically significant difference (*p* = 0.0114) was only observed in blood lead levels. Figure 2 shows the age-adjusted means (Figure 2a) and all-adjusted means (Figure 2b) of blood lead and blood cadmium levels. The age adjusted mean lead level of the infertile group (0.53 µg/dL, 95% CI: 0.46, 0.62) was significantly higher than that of the pregnant group (0.42 µg/dL, 95% CI: 0.35, 0.51) (*p* = 0.0106). Although the age adjusted mean of blood cadmium was higher in the infertile group (0.28 µg/L, 95% CI: 0.22, 0.36) than in the pregnant group (0.22 µg/L, 95% CI: 0.17, 0.28), there was no statistically significant difference (*p* = 0.1765). After adjusting for age, ethnicity, annual family income, education, marital status, smoking history, alcohol consumption, physical activity, and BMI, the mean blood lead and cadmium levels were significantly higher in the infertile group than in the pregnant group (lead:0.67 µg/dL, 95% CI: 0.53, 0.85 vs. 0.52 µg/dL, 95% CI: 0.43, 0.63, *p*-value = 0.0157; cadmium:0.45 µg/L, 95% CI: 0.32, 0.65 vs. 0.35 µg/L, 95% CI: 0.25, 0.49, *p*-value = 0.0066).

## 4. Discussion

In this cross-sectional study of US women sampled from the 2013–2014 and 2015–2016 NHANES, blood lead and cadmium levels were positively associated with self-reported infertility. The adjusted odds of infertility were approximately 2–3 times higher (per two-fold increase in blood metal levels) in infertile women than in pregnant women. Our results suggest that blood lead and cadmium levels may be a factor explaining infertility.

As far as we know, few epidemiological studies have examined the relationship between lead exposure and infertility, and some of these studies are consistent with ours [15,16,17,18]. In a case control study conducted at Odense University Hospital in Denmark, self-reported exposure to lead, cadmium, and mercury was compared among 4305 fertile and 1069 infertile couples. The results showed that exposed females had a three-fold higher risk of idiopathic infertility and a 1.7-fold higher risk of conception delay [18]. A recent study in Taiwan comparing 310 infertile women with 57 pregnant women recruited from a hospital in Taiwan showed that blood lead levels were significantly higher in infertile women than in pregnant women (mean [SD], 1.7 [0.8] vs. 1.2 [0.5] µg/dL) [17]. Guerra-Tamayo et al. (2003) investigated the blood lead levels of Mexican women and reported that those with blood lead levels above 10.0 µg/dL were five times more likely of not achieving pregnancy [16]. A study by Chang et al. [15] compared 64 ‘infertile’ women receiving care at a private infertility clinic and 83 ‘fertile’ postpartum women in terms of blood lead levels. Compared to women with blood lead levels ≤2.5 µg/dL, women with levels >2.5 µg/dL were associated with three times the risk of infertility [15].

Similar effects on infertility have been observed to a limited extent in females with cadmium exposure [18,25,31]. Danish females exposed to lead, mercury, and cadmium at the workplace were more likely to experience conception delay and idiopathic infertility relative to their controls [18]. Wang and Tian investigated the health risks of residents living near a lead-zinc mine by comparing them with controls living 40 km away from the mine [25]. They found that women living near the mine had higher urinary cadmium levels and were associated with difficulties becoming pregnant [25]. In a prospective cohort study with 501 couples desiring pregnancy and discontinuing contraception, elevated blood cadmium levels in females were significantly associated with reduced fecundity [31].

Evidence on the effects of lead and cadmium on infertility is limited and inconsistent; however, the rationale behind our observed associations can be explained by previous in vitro and animal studies [11,12,13,14,22,23,24,32,33]. Studies in mice have revealed that intraperitoneal lead and cadmium exposures affect the physiological functions of reproductive hormones via accumulation in the pituitary membrane and ovarian granulosa cells [24,33]. An analysis of the pituitary membrane showed that accumulation of metals in the pituitary membrane lowered membrane fluidity, potentially resulting in alterations in receptor binding and pituitary hormone secretion [24]. Another study revealed that accumulations of metals in ovarian granulosa cells caused a significant reduction in gonadotropin binding, altering steroidogenic enzyme activity [33]. In addition to alterations in hormone function, which serve as an important factor in the pathogenesis of infertility, lead has been associated with the dysfunction of folliculogenesis in mice [14], luteal function in monkeys [11,12], and oocyte meiosis in in vitro studies [32]. Cadmium exposure has been reported to impair oocyte maturation and fertilization in the cumulus-oocyte complexes of sheep [22] and alter the morphology and steroidogenesis of cultured human ovarian granulosa cells [23]. Analysis of cultured ovarian aspirates obtained from 41 women undergoing IVF revealed that in vitro cadmium exposure altered cell to cell contact, potentially disrupting the intercellular communication that is essential for oocyte nutrient mediation and signal transmission. In addition, cadmium exposure was also associated with decreased progesterone secretion, which is essential for embryo implantation and pregnancy maintenance [23]. Taken together, we speculate that lead and cadmium exposures may contribute to infertility through changes in hormone function and other alterations in the reproductive process.

To the best of our knowledge, this is the first population-based study that examines this relationship. Nonetheless, it is surprising to find odds in response to low concentrations of lead and cadmium levels below the reference values and biological indices for exposed workers. According to the US Centers for Disease Control and Prevention, the reference value for lead is 5 µg/dL [34], and the biological indices for blood lead and cadmium in exposed workers are 20 µg/dL and 5 µg/L, respectively [35]. However, there is no safe blood lead level in children and even low levels have shown adverse effects [36]. The adverse health effects of cadmium may occur at lower levels than previously predicted [37]. For example, such findings are supported by studies that investigated the relationship between low level heavy metal exposures and male reproductive function [38]. Epidemiological evidence, although rare, is available on the effects of low blood lead and cadmium levels and female fertility, as reported in studies by Chang et al. [15] and Lei et al. [17]. We acknowledge the fact that evidence is not sufficient to determine whether levels below 10 µg/dL affect female fertility [39]. However, our data suggest that the female reproductive system is potentially susceptible to such levels. Further population-based studies are necessary to identify reproductive toxicity at low levels of exposure to these metals.

This study has several limitations. First, due to its cross-sectional design, any casual or temporal relationships cannot be confirmed. Second, there are limitations when infertility is measured using a self-reported questionnaire. Although self-reported infertility is a useful measure, the various definitions of infertility (i.e, medical records or calendar-derived time taken trying to conceive) may affect the prevalence of measured infertility [40,41]. To eliminate the confusion between women who have never tried to conceive in the past and ‘fertile’ women who may report no difficulty conceiving, we compared metal levels between ‘infertile’ and ‘pregnant’ women in correspondence with Lei et al. [17]. Nevertheless, such comparison and classification may not be faultless and further studies should consider the various definitions. Thirdly, male factor affects infertility in approximately 17% to 28% of all cases [5]. However, the infertility questionnaire utilized in our study only addressed females, limiting our study to female infertility. Male factor infertility was not considered. Nevertheless, the relationship between male reproductive function and heavy metals has been studied extensively, whereas studies on female reproductive function are relatively rare [38]. Another limitation of this study is a small sample size, which may serve as a potential source of bias. Although efforts were taken to maximize the sample size by including all possible survey cycles that included data for infertility status, the sample population was limited to 124 females after applying the inclusion and exclusion criteria mentioned above. We therefore recommend further studies using a larger sample. Finally, although basic sociodemographic confounding variables were considered, not all possible confounding variables may have been controlled for. Some studies have also included parity [18,19], menstrual irregularities [15,17], intercourse frequency [19,20], and contraceptive use [15,18] as confounding variables. Therefore, we cannot exclude the possibility of residual confounding.

## 5. Conclusions

In conclusion, we found a significant association between low blood lead and cadmium levels and infertility in a representative sample of US adults. Although our findings require confirmation, they suggest that even low levels of heavy metals, below the current reference values, significantly affect reproductive health.

## Figures and Tables

**Table 1:** Characteristics of the study population (*n* = 124).

Characteristics	Infertile ^1^	Pregnant ^2^	*p*-Value
	*n* (%)	*n* (%)	
**Total subjects**	82 (66.13)	42 (33.87)	
Age (year)			0.0001
20–24	14 (45.16)	17 (54.84)	
25–29	15 (51.72)	14 (48.28)	
30–34	18 (69.23)	8 (30.77)	
35–39	35 (92.11)	3 (7.89)	
Ethnicity			0.4522
Non-Hispanic white	21 (67.74)	10 (32.26)	
Non-Hispanic black	23 (63.89)	13 (36.11)	
Hispanic	28 (73.68)	10 (26.32)	
Others	10 (52.63)	9 (47.37)	
Annual Family Income			0.4153
Less than $20,000	18 (60)	12 (40)	
$20,000 and over	64 (68.09)	30 (31.91)	
Education			0.9313
Less than high school	13 (65)	7 (35)	
High school graduate	18 (69.23)	8 (30.77)	
More than high school	51 (65.38)	27 (34.62)	
Marital status			0.6897
Married	46 (67.65)	22 (32.35)	
Never married	16 (59.26)	11 (40.74)	
Widowed/divorced/separated	20 (68.97)	9 (31.03)	
Smoking history			0.8661
Never smoked	16 (66.67)	8 (33.33)	
Ex-Smoker	9 (60)	6 (40)	
Current Smoker	57 (67.06)	28 (32.94)	
Alcohol consumption ^3^			0.5427
Yes	59 (67.82)	28 (32.18)	
No	23 (62.16)	14 (37.84)	
Physical Activity ^4^			0.6232
Yes	35 (68.63)	16 (31.37)	
No	47 (64.38)	26 (35.62)	
BMI (kg/m^2^)			0.0882
Underweight (<18.5)	2 (66.67)	1 (33.33)	
Normal weight (18.5–24.9)	21 (70)	9 (30)	
Overweight (25.0–29.9)	10 (43.48)	13 (56.52)	
Obesity (>30)	49 (72.06)	19 (27.94)	

^1^ ‘Infertile’ if subject responded ‘yes’ to the following question: “Have you ever attempted to become pregnant over a period of at least a year without becoming pregnant?” ^2^ ‘Pregnant’ if positive pregnancy test or self-reported pregnancy at exam ^3^ Response to the question: “In any one year, have you had at least 12 drinks of any type of alcoholic beverage?” ^4^ Response to the question: “In a typical week do you do any moderate-intensity sports, fitness, or recreational activities that cause a small increase in breathing or heart rate such as brisk walking, bicycling, swimming, or volleyball for at least 10 min continuously?”.

**Table 2:** Odds ratio (95% CI) of being infertile associated with blood lead and blood cadmium levels.

Concentrations		Prevalence, *n* (%)		Unadjusted OR	Adjusted	
		Infertile ^1^	Pregnant ^2^		Model 1 ^3^	Model 2 ^4^
*Blood lead levels*						
per 2-fold increase		82	42	2.06 (1.32–3.23)	1.81 (1.11–2.95)	2.60 (1.05–6.41)
Tertiles	Tertile 1	19 (48.72)	20 (51.28)	Reference	Reference	Reference
	Tertile 2	32 (76.19)	10 (23.81)	3.99 (1.60–9.99)	3.86 (1.32–11.26)	5.40 (1.47–19.78)
	Tertile 3	31 (72.09)	12 (27.91)	3.08 (1.26–7.54)	3.01 (0.94–9.57)	5.62 (1.13–27.90)
*Blood cadmium levels*						
per 2-fold increase				1.18 (0.83–1.66)	1.24 (0.89–1.71)	1.84 (1.07–3.15)
Tertiles	Tertile 1	24 (60.00)	16 (40.00)	Reference	Reference	Reference
	Tertile 2	29 (69.05)	13 (30.95)	1.19 (0.35–4.09)	0.84 (0.22–3.19)	1.15 (0.22–5.90)
	Tertile 3	29 (69.05)	13 (30.95)	1.59 (0.60–4.26)	1.55 (0.62–3.86)	2.47 (0.6–10.17)

^1^ ‘Infertile’ if subject responded ‘yes’ to the following question: “Have you ever attempted to become pregnant over a period of at least a year without becoming pregnant?” ^2^ ‘Pregnant’ if positive pregnancy test or self-reported pregnancy at exam ^3^ Adjusted for age only ^4^ Adjusted for age, ethnicity, annual family income, education, marital status, smoking history, alcohol consumption, physical activity and BMI.
